# Dirichlet composition distribution for compositional data with zero components: An application to fluorescence in situ hybridization (FISH) detection of chromosome

**DOI:** 10.1002/bimj.202000334

**Published:** 2021-12-16

**Authors:** Man‐Lai Tang, Qin Wu, Sheng Yang, Guo‐Liang Tian

**Affiliations:** ^1^ Department of Mathematics College of Engineering, Design & Physical Sciences Brunel University London Uxbridge United Kingdom; ^2^ Department of Statistics School of Mathematical Sciences South China Normal University, Guangzhou City Guangdong P. R. China; ^3^ Zhongshan People's Hospital Zhongshan P. R. China; ^4^ Department of Statistics and Data Science Southern University of Science and Technology Shenzhen City Guangdong P. R. China

**Keywords:** compositional data, Dirichlet distribution, EM algorithm, essential zero, gamma distribution, rounded zeros, stochastic representation

## Abstract

Zeros in compositional data are very common and can be classified into rounded and essential zeros. The rounded zero refers to a small proportion or below detection limit value, while the essential zero refers to the complete absence of the component in the composition. In this article, we propose a new framework for analyzing compositional data with zero entries by introducing a stochastic representation. In particular, a new distribution, namely the Dirichlet composition distribution, is developed to accommodate the possible essential‐zero feature in compositional data. We derive its distributional properties (e.g., its moments). The calculation of maximum likelihood estimates via the Expectation‐Maximization (EM) algorithm will be proposed. The regression model based on the new Dirichlet composition distribution will be considered. Simulation studies are conducted to evaluate the performance of the proposed methodologies. Finally, our method is employed to analyze a dataset of fluorescence in situ hybridization (FISH) for chromosome detection.

## 1 INTRODUCTION

Compositional data, which consist of vectors of positive components subject to a constant‐sum constraint (e.g., equal to 1 for proportions and 100 for percentages), record the information about the relative frequencies associated with different components of a system (Ferrers, 1886). They commonly arise in many disciplines such as the components of rocks in geology, the budget share patterns of household expenditures in economics, and the proportion of normal cells in medical research. It is noteworthy that compositional data are subject to the following two intrinsic constraints:

(a) (Bounded support constraint) Each element in the component vector must lie between 0 and 1, inclusive; and
(b) (Summation constraint) All the elements in the component vector must sum to 1.

Mosimann (1962) proposed the Dirichlet‐multinomial (DM) distribution, which is a family of discrete multivariate probability distributions on a finite support of nonnegative integers. It is noteworthy that DM is a compound probability distribution, which models counts from a multinomial distribution with a probability vector that is drawn from a Dirichlet distribution. As a result, the DM model (i.e., zero‐inflated generalized DM [ZIGDM] by Tang & Chen, 2019) is designed for count data and fails to deal with the compositional data, which are proportions or percentages, described in this article. Applications of statistical methods designed for unconstrained data to such compositional data may result in invalid inference conclusions. For instance, Pearson (1897) discussed the spurious correlation issue in compositional data analysis and concluded that the unit‐sum constraint is often intentionally ignored and the statistical methods without constraints are misused, which may eventually lead to disastrous results. Aitchison (1982) first proposed statistical methodology for the compositional data. Aitchison (1982, 1986) first introduced the logistic normal (LN) distribution as a framework for compositional data analyses. In particular, his technique assumes multivariate normality of additive log‐ratio transformed data. Since then, various researchers have extended Aitchison's approach in both theoretical and practical respects. For example, Zhang (2000) discussed various distributions for compositional data on the simplex district (e.g., the generalized Dirichlet, additive logistic, and spherical distributions). Egozue et al. (2003) introduced the isometric log‐ratio transformation.

In compositional data analysis, the presence of zero components may induce obstacles to the applications of the aforementioned distributional approaches (e.g., zero cannot be the denominator when applying the additive logistic transformation). Aitchison (1986) classified the zeros in compositional data into rounded (or trace elements) zeros and essential (or true) zeros. It is not uncommon that compositional data contain zero components due to either complete absence (i.e., essential zeros), or a small proportion or below the detection limit (i.e., rounded zeros) of certain component(s). Aitchison (1982) pointed out that the log‐ratio transformation failed to work when these zeros are denominators.

To deal with the rounded zeros, the most popular method is to replace the rounded zero(s) by a small value (i.e., zero replacement). For example, Palarea‐Albaladejo and Martín‐Fernández (2008) proposed a modified EM algorithm to replace the rounded zeros in compositional data, Hijazi (2011) developed the EM algorithm–based method to deal with rounded zeros. The nonparametric imputation approach is proposed by Martín‐Fernández et al. (2003) to handle rounded zeros.

For essential zeros, three well‐known approaches have been developed. The data amalgamation, which was proposed by Aitchison (1990), is to eliminate the components with zero elements by combining them with some other nonzero components. The second approach models the zeros separately (e.g., Aitchison, 1986; Bear & Billheimer, 2016; Zadora et al., 2010). For instance, Bear and Billheimer (2016) projected compositions with zeros onto smaller dimensional subspaces. As a result, they developed a mixture of logistic normals which successfully addresses the issues of division by zero and the log of zero. The third approach is to transform compositions into directional data on the hypersphere and develop a regression model using the Kent distribution (e.g., Kent, 1982; Scealy & Welsh, 2011), which tolerates zeros. Other methods are also investigated, such as the mixture models to eliminate the essential zeros (Stewart & Field, 2011), the latent Gaussian model (Butler & Glasbey, 2008), and the Dirichlet regression model (Tsagris & Stewart, 2018).

In clinical research, compositional data with essential zeros are not uncommon. For instance, chromosome abnormalities are considered to be the most common cause of spontaneous abortion. Fluorescence in situ hybridization (FISH) is a cytogenetic technique developed in the early 1980s (see, e.g., Langer‐Safer et al., 1982). It uses fluorescent DNA probes to target specific chromosomal locations within the nucleus, resulting in colored signals that can be detected using a fluorescent microscope. For spontaneous abortion, a damaged embryo is taken out from the gravida and the FISH technique is then employed to detect the cells which are selected randomly from the damaged embryo. Finally, the respective proportions of diploidy, triploidy, and polyploidy at chromosome 22 for those randomly chosen and tested cells are recorded for each embryo. Obviously, the observations are compositional data (i.e., total sum is equal to one). For example, an observation of (0.2,0.3,0.5) means 20%, 30%, and 30% of the selected cells are chromosome diploidy, chromosome triploidy, and chromosome polyploidy, respectively. The FISH data reported in the Supporting Information are the compositional observations of 51 embryos from the curettage operation in Zhongshan People's Hospital in Mainland China. The age of each gravida is also reported. It is noteworthy that nearly 80% (i.e., 40 out of 51) of the embryos demonstrate purely normal chromosomes (i.e., compositional observation being (1,0,0)). Most importantly, none of the aforementioned approaches are suitable for our FISH data, which motivate the present article.

The rest of this paper is organized as follows. In Section 2, we introduce a new stochastic representation (SR) for compositional data with zero components and the new Dirichlet composition distribution (DCD) is defined. Likelihood‐based methods for parameter estimation and confidence intervals construction without covariates will be provided in Section 3. Regression model analysis based on the distribution will be considered in Section 4. Simulation studies will be conducted to examine the performance of our proposed methods in Section 5. We will revisit and analyze the FISH dataset in Section 6. A brief discussion will be presented in Section 7. Some technical details are included in the Appendix.

## 2 NEW DEFINITION OF COMPOSITIONAL RANDOM VECTOR AND THE DCD

In this section, we introduce a new definition of a compositional random vector which can be adopted for modeling the compositional data. The definition is proposed based on SR. We then introduce the DCD by assuming the base vector following independent Gamma distributions.

### 2.1 Definition of a compositional random vector

To model the zero elements in the compositional data, we employ the SR to establish the definition of a compositional random vector.

Definition 1 (Compositional random vector). A random vector $X={\left(X_{1},\text{…},X_{m}\right)}^{\;⊤\;}$ is said to be an m-dimensional compositional random vector if

(1) $$X\;\overset{d}{=}\;\frac{Z\circ Y}{Z^{\;⊤\;}Y}$$

with $Z={\left(Z_{1},\text{…},Z_{m}\right)}^{\;⊤\;}$ being the indicator vector (i.e., $Z_{j}$ = 0 or 1 for j=1,…,m) such that $\sum_{j=1}^{m}Z_{j}$ ≠ 0, and $y={\left(Y_{1},\text{…},Y_{m}\right)}^{\;⊤\;}$ being the base vector with each element being positive random variable (i.e., $Y_{j}\in \left(0,+\infty \right)$, for j=1,…,m), “$\overset{d}{=}$” meaning the random variables on both sides have the same distribution, $Z\circ Y={\left(Z_{1}Y_{1},\text{…},Z_{m}Y_{m}\right)}^{\;⊤\;}$ and $Z^{\;⊤\;}Y=\sum_{i=1}^{n}Z_{i}Y_{i}$.

The indicator vector Z provides the possibility of zero entries in the distribution with $Z_{j}=0$ meaning the jth component in X being zero. The base vector Y carries the quantitative information. It can be any positive vector and determines the nonzero components in X.

### 2.2 Definition of DCD

In the compositional random vector, if we let Z be the m‐dimensional independent Bernoulli random variables by excluding the point 00, and Y be the independent Gamma random variable with different shape parameters $\alpha_{i}$ but identical rate parameter β, we can define a new distribution called DCD. Since the rate parameter β will be eliminated in the SR of the compositional random vector, β is unidentifiable in the distribution. Without loss of generality, we assume β=1. That is, for each $Y={\left(Y_{1},\text{…},Y_{m}\right)}^{\;⊤\;}$, we have ${\left\{Y_{i}\right\}}_{i=1}^{m}\overset{\mathrm{ind}}{∼}\text{Gamma}\left(\alpha_{i},1\right)$.

Definition 2 (DCD). A compositional random vector $X\overset{d}{=}\frac{Z\circ Y}{Z^{\;⊤\;}Y}$ is said to follow the DCD, denoted by DCD(p,α), if Z⊥⊥Y and

(2) $$\begin{array}{ccc} \mathrm{Pr}(Z=z) & = & \frac{\prod_{j=1}^{m}{\left(1-p_{j}\right)}^{z_{j}}p_{j}^{1-z_{j}}}{1-p_{1}\cdots p_{m}}, \end{array}$$

and

(3) $$\begin{array}{ccc} f(Y=y) & = & \prod_{i=1}^{m}\frac{e^{y_{i}}y_{i}^{\alpha_{i}-1}}{\Gamma (\alpha_{i})}, \end{array}$$

where $p={\left(p_{1},\text{…},p_{m}\right)}^{⊤}$ contains the parameters of the indicator vector and $\alpha ={\left(\alpha_{1},\text{…},\alpha_{m}\right)}^{⊤}$ contains the parameters of the base vector with ${\left\{0\leq p_{j}<1,\alpha_{j}>0\right\}}_{j=1}^{m}$.

The probability density function of X is then given by

(4) $$\begin{array}{c} f\left(x\right)=\frac{\prod_{j=1}^{m}p_{j}^{1-z_{j}}{\left(1-p_{j}\right)}^{z_{j}}}{1-p_{1}\cdots p_{m}}\times \left[\frac{\Gamma (\alpha_{J}^{*})}{\prod_{{}_{j\in J}}\Gamma \left(\alpha_{j}\right)}\prod_{{}_{j\in J}}x_{j}^{\alpha_{j}-1}\right], \end{array}$$

where $z_{j}=I\left(x_{j}>0\right),j=1,\text{…},m$ and $\alpha_{J}^{*}=\sum_{j\in J}\alpha_{j}$, J is the subset of the index with x being positive (i.e., $x_{j}>0$ for any j∈J and $x_{j}=0$ for j∉J). (For more details of the probability density function, refer to Appendix A.1.)

Remark 1 It is clear that $p_{j}\neq 1$ for j=1,…,m. As $\mathrm{Pr}\left(Z_{j}=0\right)=p_{j}=1$ implies that the element in the jth column must be 0, we can simply delete the jth column and the remaining m−1 columns still form a compositional random vector.

Remark 2 If $p_{j}=0$ for j=1,…,m, it means $Z_{j}=1$, we have DCD(00,α)=Dirichlet$\left(\alpha_{1},\text{…},\alpha_{m}\right)$. That is, the well‐known Dirichlet distribution is a special case of DCD(p,α).

Remark 3 We here suppose Z follows the zero‐truncated multivariate Bernoulli distribution. Due to SR in (1), the denominator $Z^{\;⊤\;}Y=\sum_{i=1}^{n}Z_{i}Y_{i}$ must be nonzero; therefore, $Z_{1},\text{…},Z_{m}$ are not independent as they cannot be 0 at the same time. That is, ${\left\{Z_{j}\right\}}_{j=1}^{m}$ follow the independent Bernoulli distributions but exclude the point 00.

### 2.3 Mixed moments and moment generating function

If X∼DCD(p,α), then the following results can be easily shown:

(5) $$\begin{array}{ccc} E(X) & = & \begin{array}{c} \sum_{z}\frac{\prod_{j=1}^{m}p_{j}^{1-z_{j}}{\left(1-p_{j}\right)}^{z_{j}}}{1-p_{1}\cdots p_{m}}\times {\left(0\;\;\;0_{K},\frac{\alpha_{J}}{\alpha_{J}^{*}}\right)}^{⊤}, \end{array}\\ \text{Cov}(X,X) & = & \begin{array}{c} \sum_{z}\frac{\prod_{j=1}^{m}p_{j}^{1-z_{j}}{\left(1-p_{j}\right)}^{z_{j}}}{1-p_{1}\cdots p_{m}}\times \text{Cov}\left(X|Z,X|Z\right), \end{array} \end{array}$$

(6) $$\begin{array}{c} E\left(\prod_{j\in J}X_{j}^{r_{j}}\right)=\frac{B(\sum_{j\in J}\alpha_{j}+\sum_{j\in J}r_{j})}{B(\sum_{j\in J}\alpha_{j})};\;E\left(\prod_{\exists j\notin J}X_{j}^{r_{j}}\right)=0, \end{array}$$

where K is the subset with x being 0 (i.e., K={1,…,m}∖J), ${\left(0\;\;\;0_{K},\frac{\alpha_{J}}{\alpha_{J}^{*}}\right)}^{⊤}$= ${\left(e_{i}\right)}_{m\times 1}$, and $\text{Cov}\left(X,X\right)={\left(v_{ij}\right)}_{m\times m}$

(7) $$\begin{array}{ccc} e_{i}= & 0; & \text{if}\;i\in K;\\ e_{i}= & \frac{\alpha_{i}}{\alpha_{J}^{*}}; & \text{if}\;i\in J;\\ v_{ii}= & 0; & \text{if}\;i\in K;\\ v_{ii}= & \frac{\alpha_{i}\left(\alpha_{J}^{*}-\alpha_{i}\right)}{{\left(\alpha_{J}^{*}\right)}^{2}\left(\alpha_{J}^{*}+1\right)}, & \text{if}\;i\in J;\\ v_{ij}= & 0; & \text{if}\;i\in K\;\text{or}\;j\in K;\\ v_{ij}= & -\frac{\alpha_{i}\alpha_{j}}{{\left(\alpha_{J}^{*}\right)}^{2}\left(\alpha_{J}^{*}+1\right)}, & \text{if}\;i,j\in J. \end{array}$$

## 3 Statistical inference without covariates

In this section, we present statistical inferences based on data without covariates, which include the maximum likelihood estimates (MLEs) calculation in Section 3.1 and the confidence interval construction in Section 3.2.

### 3.1 Maximum likelihood estimates for target parameters

Suppose the n observations are $\left\{x_{1},\text{…},x_{n}\right\}$, where $x_{i}={\left(x_{i1},\text{…},x_{im}\right)}^{\;⊤\;}$ and m is the number of dimensions. Without loss of information, we assume that there are zero entries in the first k observations, that is $\min(x_{i})=0$ for i=1,…,k, and $\min(x_{i})>0$ for i=k+1,…,n, 0≤k≤m. We have $J_{i}={\left(1,2,\cdots ,m\right)}^{⊤}$ when i≥k. Therefore, the observed likelihood function is given by

(8) $$\begin{array}{ccc} L(p,\alpha |Y_{\mathrm{obs}}) & = & \left\{\prod_{i=1}^{n}\frac{\prod_{j=1}^{m}p_{j}^{1-z_{ij}}{\left(1-p_{j}\right)}^{z_{ij}}}{1-p_{1}\cdots p_{m}}\right\}\times \left\{\prod_{i=1}^{k}\left[\frac{\Gamma (\alpha_{J_{i}}^{*})}{\prod_{{}_{j\in J_{i}}}\Gamma \left(\alpha_{j}\right)}\prod_{{}_{j\in J_{i}}}x_{ij}^{\alpha_{j}-1}\right]\right\}\\ & & \times \left\{\prod_{i=k+1}^{n}\left[\frac{\Gamma (\sum_{j=1}^{m}\alpha_{j})}{\prod_{j=1}^{m}\Gamma \left(\alpha_{j}\right)}\prod_{j=1}^{m}x_{ij}^{\alpha_{j}-1}\right]\right\}, \end{array}$$

where $J_{i}$ denotes the index set of those positive elements in each $x_{i}$ (i.e., $x_{ij}>0$ if $j\in J_{i}$). Here, the indicator variable Z can be observed via the observation $x_{i}$ as

(9) $$Z_{ij}|\left(X_{i}=x_{i}\right)=\left\{\begin{array}{ccc} 0, & & \text{if}\;x_{ij}=0,\\ 1, & & \text{if}\;x_{ij}>0. \end{array}\right.$$

Observing that $Z_{j}=1$ is equivalent to $j\in J_{i}$. we have

(10) $$\begin{array}{ccc} l(p,\alpha |Y_{\mathrm{obs}}) & = & \left\{\sum_{i=1}^{n}\left[\left(1-z_{ij}\right)\log p_{j}+z_{ij}\log\left(1-p_{j}\right)-\log\left(1-p_{1}\cdots p_{m}\right)\right]\right\}\\ & & +\sum_{i=1}^{k}\left\{\log\Gamma \left(\alpha_{J_{i}}^{*}\right)+\sum_{j\in J_{i}}\left[\left(\alpha_{j}-1\right)\log x_{ij}-\log\Gamma \left(\alpha_{j}\right)\right]\right\}\\ & & +\sum_{i=k+1}^{n}\left\{\log\Gamma \left(\sum_{j=1}^{m}\alpha_{j}\right)+\sum_{j=1}^{m}\left[\left(\alpha_{j}-1\right)\log x_{ij}-\log\Gamma \left(\alpha_{j}\right)\right]\right\}. \end{array}$$

Instead of obtaining the MLEs of the parameters $p={\left(p_{1},\text{…},p_{m}\right)}^{\;⊤\;}$ and $\alpha ={\left(\alpha_{1},\text{…},\alpha_{m}\right)}^{\;⊤\;}$ via solving the solutions to the system of equations $\frac{\partial l(p,\alpha |Y_{\mathrm{obs}})}{\partial p}$ = 00 and $\frac{\partial l(p,\alpha |Y_{\mathrm{obs}})}{\partial \alpha }$ = 00, we consider the EM algorithm. Motivated by the SR, we introduce the base vectors ${\left\{y_{i}\right\}}_{i=1}^{n}$ and s as missing data, where s denotes the number of unobserved 00 to make the components in Z being independent. In fact, Z are independent Bernoulli variables which exclude the outcome of 00. Therefore, $\left\{{\underset{︸}{0\;\;\;0,\cdots ,0\;\;\;0}}_{s},{\left\{z_{i}\right\}}_{i=1}^{n}\right\}$ are the complete observations and the likelihood function based on the complete data is given by

(11) $$\begin{array}{ccc} L_{com}\left(p,\alpha |Y_{\mathrm{com}}\right) & = & {\left(p_{1}\cdots p_{m}\right)}^{s}\times \left\{\prod_{i=1}^{n}\prod_{j=1}^{m}\left[p_{j}^{1-z_{ij}}{\left(1-p_{j}\right)}^{z_{ij}}\times \frac{e^{y_{ij}}y_{ij}^{\alpha_{j}-1}}{\Gamma (\alpha_{j})}\right]\right\}, \end{array}$$

and the log‐likelihood function of the complete data likelihood function is

(12) $$\begin{array}{ccc} l_{com}\left(p,\alpha |Y_{\mathrm{com}}\right) & = & s\sum_{j=1}^{m}\log p_{j}+\sum_{i=1}^{n}\sum_{j=1}^{m}\left[z_{ij}\log\left(1-p_{j}\right)+\left(1-z_{ij}\right)\log p_{j}\right]\\ & + & \left\{\sum_{i=1}^{n}\sum_{j=1}^{m}\left[-y_{ij}+\left(\alpha_{j}-1\right)\log y_{ij}-\log\Gamma \left(\alpha_{j}\right)\right]\right\}. \end{array}$$

The M‐step is to solve the following equations, for j=1,…,m:

(13) $$\left\{\begin{array}{ccc} \frac{\partial l_{com}\left(p,\alpha |Y_{\mathrm{com}}\right)}{\partial p_{j}} & = & \frac{s}{p_{j}}+\sum_{i=1}^{n}\left(\frac{1-z_{ij}}{p_{j}}-\frac{z_{ij}}{1-p_{j}}\right)=0,\\ \frac{\partial l_{com}\left(p,\alpha |Y_{\mathrm{com}}\right)}{\partial \alpha_{j}} & = & \sum_{i=1}^{n}\left[\log y_{ij}-\psi \left(\alpha_{j}\right)\right]=0, \end{array}\right.$$

where ψ denotes the digamma function with $\psi \left(\alpha \right)=\frac{d\log(\Gamma (\alpha ))}{d\alpha }=\frac{\Gamma^{\prime }\left(\alpha \right)}{\Gamma (\alpha )}$. For $p_{j},j=1,\text{…},m$, we have

(14) $$\begin{array}{c} p_{j}=1-\frac{\sum_{i=1}^{n}z_{ij}}{n+s}. \end{array}$$

However, there are no closed‐form solutions for $\alpha_{j}$s and we will use the following Newton–Raphson iterative algorithm to find the MLEs of αs.

(15) $$\begin{array}{c} \alpha_{j}^{\left(t+1\right)}=\alpha_{j}^{\left(t\right)}-{\left[\frac{\partial l_{com}^{2}\left(p,\alpha |Y_{\mathrm{com}}\right)}{\partial \alpha_{j}^{2}}\right]}^{-1}\times \frac{\partial l_{com}\left(p,\alpha |Y_{\mathrm{com}}\right)}{\partial \alpha_{j}}, \end{array}$$

where $\frac{\partial l_{com}^{2}\left(p,\alpha |Y_{\mathrm{com}}\right)}{\partial \alpha_{j}^{2}}=-n\phi \left(\alpha_{j}\right)$ with $\phi \left(\alpha_{j}\right)=\frac{d^{2}\log\left(\Gamma \left(\alpha \right)\right)}{d\alpha^{2}}=\frac{d\psi (\alpha )}{d\alpha }$ being the trigamma function.

To obtain the E step, we have the following theorem and the proof is presented in Appendix A.2.

Theorem 1 The conditional expectation of $\log y_{ij}$ given x is as follows:

(16) $$E\left(\log y_{j}|x\right)=\left\{\begin{array}{ccc} \psi (\alpha_{j}), & & \text{if}\;x_{j}=0,\\ \psi \left(\alpha_{J_{i}}^{*}\right)+\log x_{j} & & \text{if}\;x_{j}>0. \end{array}\right.$$

The E‐step is to replace the missing data by the following conditional expectations:

(17) $$\begin{array}{ccc} E(s) & = & \frac{np_{1}\cdots p_{m}}{1-p_{1}\cdots p_{m}}\\ E(\log y_{j}|x) & = & \left\{\begin{array}{ccc} \psi (\alpha_{j}), & & \text{if}\;x_{j}=0,\\ \psi \left(\alpha_{J_{i}}^{*}\right)+\log x_{j} & & \text{if}\;x_{j}>0. \end{array}\right. \end{array}$$

Here, we can consider the initial values of parameters being $\alpha^{0}={\left(1,\cdots ,1\right)}^{\;⊤\;}$ in the EM algorithm. The above steps (i.e., E‐ and M‐steps) are repeated until a certain convergence condition is achieved. For instance, if the difference between two successive log‐likelihood values is less than the prespecified value 0.001, the algorithm stops after 100–150 iterations.

### 3.2 Confidence interval construction

In this section, we will consider the construction of confidence intervals for target parameters using the bootstrap method. It is noted that the value of $p_{j}$ must be restricted within the interval [0,1]. However, Wald‐type confidence intervals may produce upper (or lower) limit larger (or less) than 1 (or 0). It is noteworthy that the MLE of p obtained via our proposed EM algorithm always lies between 0 and 1. As a result, we apply the bootstrap method to create the bootstrap confidence interval (CI) for any arbitrary function of θ=(p,α), denoted by ϑ=h(θ). Briefly, based on the observations, we can independently generate ${\left\{y_{i}\right\}}_{i=1}^{n}$ with each $\left\{y_{i}\right\}$ is randomly selected from the n observations with replacement. Having obtained $Y_{\mathrm{obs}}^{*}=\left\{y_{1}^{*},\text{…},y_{n}^{*}\right\}$, we can calculate the parameter estimates ${\hat{\theta }}^{*}$ and get the bootstrap replication ${\hat{\vartheta }}^{*}=h\left({\hat{\theta }}^{*}\right)$. Independently repeating this process B times, we obtain B replications ${\left\{{\hat{\vartheta }}_{g}^{*}\right\}}_{g=1}^{B}$. The bootstrap CI of ϑ can be constructed by $\left[\vartheta_{{}_{L}},\vartheta_{{}_{U}}\right]$, where $\vartheta_{{}_{L}}$ and $\vartheta_{{}_{U}}$ are the 100(α/2) and 100(1 − α/2) percentiles of ${\left\{{\hat{\vartheta }}_{g}^{*}\right\}}_{g=1}^{B}$, respectively.

## 4 STATISTICAL INFERENCE WITH COVARIATES

In this section, we will show how to formulate the regression model for the target parameters and how to obtain the MLEs of the coefficients in the regression model. Let the covariates of each observation be denoted by $\left\{v_{i},w_{i}\right\}$, i=1,⋯,n. We consider the following regression models:

(18) $$\left\{\begin{array}{ccc} X_{i} & \overset{\mathrm{ind}}{∼} & \text{DCD}(p_{i},\alpha_{i}),\;i=1,\text{…},n,\\ \log\left(\frac{p_{ij}}{1-p_{ij}}\right) & = & v_{i}^{\;⊤\;}\beta_{j},\;\text{and}\\ \log(\alpha_{ij}) & = & w_{i}^{\;⊤\;}\gamma_{j},\;j=1,\text{…},m. \end{array}\right.$$

Let $s_{i}$ denote the number of supplementary 00 to make the elements in $z_{i}$ being independent, where i=1,…,n. Obviously, $s_{1},\text{…},s_{n}$ are missing data with $s_{i}=1$ being equivalent to $Z_{i1}=\cdots Z_{im}=0$ and $\mathrm{Pr}\left(s_{i}=1\right)=p_{i1}\cdots p_{im}$. Thus, the complete likelihood function is given by

(19) $$\begin{array}{ccc} L_{1}\left(\beta ,\gamma |Y_{\mathrm{com}}\right) & = & \prod_{i=1}^{n}\prod_{j=1}^{m}\left[p_{ij}^{1-z_{ij}}{\left(1-p_{ij}\right)}^{z_{ij}}{\left(p_{i1}\cdots p_{im}\right)}^{s_{i}}\times \frac{e^{-y_{ij}}y{}_{ij}^{\alpha_{ij}-1}}{\Gamma (\alpha_{ij})}\right]\\ & = & \prod_{i=1}^{n}\prod_{j=1}^{m}\left[{\left(\frac{e^{v_{i}^{\;⊤\;}\beta_{j}}}{1+e^{v_{i}^{\;⊤\;}\beta_{j}}}\right)}^{1-z_{ij}}{\left(\frac{1}{1+e^{v_{i}^{\;⊤\;}\beta_{j}}}\right)}^{z_{ij}}{\left(\prod_{l=1}^{m}\frac{e^{w_{i}^{\;⊤\;}\beta_{l}}}{1+e^{w_{i}^{\;⊤\;}\beta_{l}}}\right)}^{s_{i}}\times \frac{e^{-y_{ij}}y{}_{ij}^{e^{w_{i}^{\;⊤\;}\gamma_{j}}-1}}{\Gamma (e^{w_{i}^{\;⊤\;}\beta_{j}})}\right]. \end{array}$$

Or, the log‐likelihood function is

(20) $$\begin{array}{ccc} l_{1}\left(\beta ,\gamma |Y_{\mathrm{com}}\right) & = & \sum_{i=1}^{n}\sum_{j=1}^{m}\left[\left(1-z_{ij}+s_{i}\right)\log p_{ij}+z_{ij}\log\left(1-p_{ij}\right)\right]\\ & & +\sum_{i=1}^{n}\sum_{j=1}^{m}\left[-y_{ij}+\left(\alpha_{ij}-1\right)\log\left(y_{ij}\right)-\log\left(\Gamma \left(\alpha_{ij}\right)\right)\right]\\ & = & \sum_{i=1}^{n}\sum_{j=1}^{m}\left\{\left(1+s_{i}\right)\left[v_{i}^{\;⊤\;}\beta_{j}-\log\left(1+e^{v_{i}^{\;⊤\;}\beta_{j}}\right)\right]-z_{ij}v_{i}^{\;⊤\;}\beta_{j}\right\}\\ & & +\sum_{i=1}^{n}\sum_{j=1}^{m}\left[-y_{ij}+\left(e^{w_{i}^{\;⊤\;}\gamma_{j}}-1\right)\log\left(y_{ij}\right)-\log\left(\Gamma \left(e^{w_{i}^{\;⊤\;}\gamma_{j}}\right)\right)\right]\\ & = & \sum_{i=1}^{n}\sum_{j=1}^{m}\left[\left(1+s_{i}-z_{ij}\right)v_{i}^{\;⊤\;}\beta_{j}-\left(1+s_{i}\right)\log\left(1+e^{v_{i}^{\;⊤\;}\beta_{j}}\right)\right]\\ & & +\sum_{i=1}^{n}\sum_{j=1}^{m}\left[-y_{ij}+\left(e^{w_{i}^{\;⊤\;}\gamma_{j}}-1\right)\log\left(y_{ij}\right)-\log\left(\Gamma \left(e^{w_{i}^{\;⊤\;}\gamma_{j}}\right)\right)\right]. \end{array}$$

The MLEs of the regression coefficients are the solution to the following equations:

(21) $$\begin{array}{c} \frac{\partial l_{1}\left(\beta ,\gamma |Y_{com}\right)}{\partial \beta }=0\;\;\;0\;\text{and}\;\frac{\partial l_{1}\left(\beta ,\gamma |Y_{com}\right)}{\partial \gamma }=0\;\;\;0. \end{array}$$

It is obvious that there is no closed‐form solution to (21). Here, we use the Newton–Raphson algorithm to calculate the MLEs, and the iterations are given by

(22) $$\begin{array}{ccc} \beta_{j}^{\left(t+1\right)} & = & \beta_{j}^{\left(t\right)}-{\left[\frac{\partial l_{1}^{2}\left(\beta ,\gamma |Y_{\mathrm{com}}\right)}{\partial \beta_{j}\beta_{j}^{\;⊤\;}}\right]}^{-1}\times \frac{\partial l_{1}\left(\beta ,\gamma |Y_{\mathrm{com}}\right)}{\partial \beta_{j}}\;\text{and}\\ \gamma_{j}^{\left(t+1\right)} & = & \gamma_{j}^{\left(t\right)}-{\left[\frac{\partial l_{1}^{2}\left(\beta ,\gamma |Y_{\mathrm{com}}\right)}{\partial \gamma_{j}\gamma_{j}^{\;⊤\;}}\right]}^{-1}\times \frac{\partial l_{1}\left(\beta ,\gamma |Y_{\mathrm{com}}\right)}{\partial \gamma_{j}},\;\;j=1,\text{…},m. \end{array}$$

The first and negative second partial derivatives of the complete‐data log‐likelihood function are given by

(23) $$\begin{array}{ccc} \frac{\partial l_{1}\left(\beta ,\gamma |Y_{\mathrm{com}}\right)}{\partial \beta_{j}} & = & \sum_{i=1}^{n}\left[\left(1-z_{ij}-p_{ij}\right)v_{i}+s_{i}\left(1-p_{ij}\right)v_{i}\right]\\ & = & V^{\;⊤\;}\left[\left(1\;\;\;1-z_{\left(j\right)}-p_{\left(j\right)}\right)+\left(1\;\;\;1-p_{\left(j\right)}\right)\circ s\right],\\ \frac{\partial l_{1}\left(\beta ,\gamma |Y_{\mathrm{com}}\right)}{\partial \gamma_{j}} & = & \sum_{i=1}^{n}\left[\alpha_{ij}\log y_{ij}-\psi \left(\alpha_{ij}\right)\alpha_{ij}\right]w_{i}=W^{\;⊤\;}\left[\alpha_{\left(j\right)}\circ \left(\log\left(y_{\left(j\right)}\right)-\psi \left(\alpha_{\left(j\right)}\right)\right)\right],\\ -\frac{\partial^{2}l_{1}\left(\beta ,\gamma |Y_{\mathrm{com}}\right)}{\partial \beta_{j}\partial \beta_{j}^{\;⊤\;}} & = & \sum_{i=1}^{n}\left[p_{ij}\left(1-p_{ij}\right)\left(1+s_{i}\right)v_{i}v_{i}^{\;⊤\;}\right]\\ & = & V^{\;⊤\;}\text{diag}[p_{\left(j\right)}\circ \left(1\;\;\;1-p_{\left(j\right)}\right)\circ \left(1\;\;\;1+s\right]V\\ & \;\hat{=}\; & J_{\mathrm{com}}\left(\beta_{j}\right),\;\text{and}\\ -\frac{\partial^{2}l_{1}\left(\beta ,\gamma |Y_{\mathrm{com}}\right)}{\partial \gamma_{j}\partial \gamma_{j}^{\;⊤\;}} & = & \sum_{i=1}^{n}\left[\alpha_{ij}\log y_{ij}-\phi \left(\alpha_{ij}\right)\alpha_{ij}^{2}-\psi \left(\alpha_{ij}\right)\alpha_{ij}\right]w_{i}w_{i}^{\;⊤\;}\\ & = & -W^{\;⊤\;}\text{diag}\left[(\alpha_{\left(j\right)}\circ \log\left(y_{\left(j\right)}\right)-\Phi \left(\alpha_{\left(j\right)}\right)\circ \alpha_{\left(j\right)}^{2}-\Psi \left(\alpha_{\left(j\right)}\right)\circ \alpha_{\left(j\right)})\right]W\\ & \;\hat{=}\; & J_{\mathrm{com}}\left(\gamma_{j}\right), \end{array}$$

where

(24) $$\begin{array}{ccccccccc} V & = & {\left(v_{1},\text{…},v_{n}\right)}^{\;⊤\;}, & z_{\left(j\right)} & = & {\left(z_{1j},\text{…},z_{nj}\right)}^{\;⊤\;}, & p_{\left(j\right)} & = & {\left(p_{1j},\text{…},p_{nj}\right)}^{\;⊤\;},\\ W & = & {\left(w_{1},\text{…},w_{n}\right)}^{\;⊤\;} & s & = & {\left(s_{1},\text{…},s_{n}\right)}^{\;⊤\;}, & \alpha_{\left(j\right)} & = & {\left(\alpha_{1j},\text{…},\alpha_{nj}\right)}^{\;⊤\;},\\ y_{\left(j\right)} & = & {\left(y_{1j},\text{…},y_{nj}\right)}^{\;⊤\;}, & j & = & 1,\text{…},m. & & & \; \end{array}$$

Here, $J_{\mathrm{com}}\left(\beta_{i}\right)$ and $J_{\mathrm{com}}\left(\gamma_{i}\right)$ are actually the complete‐data Fisher information matrices associated with the parameter vectors $\beta_{j}$ and $\gamma_{j}$, respectively, which depend on neither the observed data nor the latent/missing data.

To obtain the MLEs of the parameter vectors $\beta_{j}$ and $\gamma_{j}$ in the presence of missing data (i.e., $s_{1},\text{…},s_{n}$), we introduce the EM algorithm. Briefly, the M‐step is to separately calculate the MLEs of $\beta_{j}$ and $\gamma_{j}$ via Newton–Raphson algorithms as follows:

(25) $$\left\{\begin{array}{ccc} \beta_{j}^{\left(t+1\right)} & = & \beta_{j}^{\left(t\right)}+J_{\mathrm{com}}^{-1}\left(\beta_{j}^{\left(t\right)}\right)V^{\;⊤\;}\left[\left(1\;\;\;1-z_{\left(j\right)}-p_{\left(j\right)}\right)+\left(1-p_{\left(j\right)}\right)s\right],\;\text{and}\\ \gamma_{j}^{\left(t+1\right)} & = & \gamma_{j}^{\left(t\right)}+J_{\mathrm{com}}^{-1}\left(\gamma_{i}^{\left(t\right)}\right)W^{\;⊤\;}\left[\alpha_{\left(j\right)}\circ \log\left(y_{\left(j\right)}\right)-\psi \left(\alpha_{\left(j\right)}\right)\right],\;j=1,\text{…},m. \end{array}\right.$$

The E‐step is to replace $s_{i}$ in (25) by their conditional expectations, that is,

(26) $$\begin{array}{ccc} E(s_{i}|Y_{\mathrm{obs}},\beta ,\gamma ) & = & \left(\prod_{j=1}^{m}p_{ij}\right)/\left(1-\prod_{j=1}^{m}p_{ij}\right), \end{array}$$

(27) $$\begin{array}{ccc} E(\log y_{ij}|Y_{\mathrm{obs}},\beta ,\gamma ) & \overset{\left(6\right)}{=} & \left\{\begin{array}{ccc} \psi (\alpha_{ij}), & & \text{if}\;x_{ij}=0,\\ \psi \left(\sum_{j\in J_{i}}\alpha_{ij}\right)+\log x_{ij}, & & \text{if}\;x_{ij}>0 \end{array}\right., \end{array}$$

where

(28) $$\begin{array}{ccc} p_{ij} & = & \frac{e^{v_{i}^{\;⊤\;}\beta_{j}}}{1+e^{v_{i}^{\;⊤\;}\beta_{j}}}\;\text{and}\;\\ \alpha_{ij} & = & e^{w_{i}^{\;⊤\;}\gamma_{j}},\;j=1,\text{…},m;\;i=1,\text{…},n. \end{array}$$

Remark 4 The calculation of coefficients usually works well when the dimension is not large. However, the Newton–Raphson algorithm may fail to work when the dimension is high due to the Jacobian (i.e., $J_{com}\left(\beta \right)$) tending to be 0 in some iterations. Therefore, studies with a large number of covariates should be carefully handled in order to get reliable estimates. This will be an interesting and practical topic for future research.

## 5 HYPOTHESIS TEST

We are usually interested in whether some of the coefficients/parameters are equal to zero. In this section, we will consider the *likelihood ratio test* (LRT) for the following hypotheses:

(29) $$H_{0}:\;\beta_{i1}=\cdots =\beta_{ir}=\gamma_{j1}=\cdots =\gamma_{jt}=0\;\;\;0\;\text{against}\;H_{1}:\;\text{not}\;\;H_{0},$$

where $i_{1},\text{…},i_{r}$ satisfy $1\leq i_{1}<\cdots <i_{r}\leq L_{1}$, $1\leq j_{1}<\cdots <jt\leq L_{2}$, and $L_{1}$ and $L_{2}$ are the number of covariates related to p and α, respectively. The LRT statistic is then given by

(30) $$T=-2[\ell \left({\hat{\beta }}_{0},{\hat{\gamma }}_{0}|Y_{\mathrm{obs}}\right)-\ell \left(\hat{\beta },\hat{\gamma }|Y_{\mathrm{obs}}\right)],$$

where $\left({\hat{\beta }}_{0},{\hat{\gamma }}_{0}\right)$ are the constrained MLEs of (β,γ) under $H_{0}$ and $\hat{\beta },\hat{\gamma }$ are the unconstrained MLE of (β,γ). Under the null hypothesis $H_{0}$, the p‐value is given by

(31) $$p=\mathrm{Pr}\left(T>t|H_{0}^{\prime }\right)=\mathrm{Pr}\left(\chi^{2}\left(\nu \right)>t\right),$$

where t is the observed value of T and $\chi^{2}\left(\nu \right)$ is the chi‐square distribution with ν = r + t degrees of freedom.

## 6 SIMULATION STUDIES

To evaluate the performance of the proposed statistical methods of DCD, we first investigate the accuracy of point estimates and confidence interval estimates for different parameter settings via simulation studies. We then conduct simulation studies for the regression model. The MLEs of parameters, standard deviation, and confidence intervals are presented. We will compare the ZIGDM model proposed by Tang and Chen (2019) with our proposed DCD model. Finally, simulation results for hypothesis testing are presented. In this section, all statistical computations are implemented in R.

### 6.1 Accuracy of point and interval estimates

For the m‐dimensional compositional data, there are 2m parameters in the DCD (i.e., the m‐dimensional parameter $p={\left(p_{1},\text{…}p_{m}\right)}^{⊤}$ and m‐dimensional parameter $\alpha ={\left(\alpha_{1},\text{…}\alpha_{m}\right)}^{⊤}$).

We consider two cases, m=2 and m=3, to evaluate the accuracy of point and confidence interval estimates. When m=2, we set (p,α)=(0.1,0.2,3,4) or (0.1,0.4,2,1). When m=3, we set (p,α)=(0.1,0.3,0.2,3,2,4) or (0.2,0.2,0.3,2,1,3). For each parameter configuration, we generate ${\left\{y_{i}\right\}}_{i=1}^{n}∼\text{DCD}\left(p,\alpha \right)$ with n=100,300,500, and calculate the MLEs via the EM algorithm and the 95% bootstrap CIs with a significance level α=0.05 with B=1000. The MLEs of parameters, the width, and coverage probability of the bootstrap confidence interval are presented in Tables 1 and 2 for m=2 and m=3, respectively.

**TABLE 1:** MLEs and bootstrap confidence intervals of parameters when m=2

	n=100			n=300			n=500		
True value	MLE	Width	CP	MLE	Width	CP	MLE	Width	CP
$p_{1}$ = 0.1	0.101	0.128	0.940	0.101	0.075	0.950	0.100	0.058	0.955
$p_{2}$ = 0.2	0.199	0.163	0.939	0.199	0.094	0.955	0.199	0.073	0.958
$\alpha_{1}$ = 3	3.131	2.072	0.916	3.049	1.119	0.937	3.024	0.852	0.952
$\alpha_{2}$ = 4	4.191	2.823	0.910	4.068	1.516	0.930	4.032	1.157	0.946
$p_{1}$ = 0.1	0.099	0.145	0.935	0.100	0.085	0.935	0.100	0.067	0.950
$p_{2}$ = 0.4	0.398	0.198	0.960	0.399	0.115	0.941	0.399	0.089	0.945
$\alpha_{1}$ = 2	2.136	1.711	0.890	2.060	0.889	0.918	2.040	0.682	0.937
$\alpha_{2}$ = 1	1.053	0.748	0.896	1.025	0.399	0.919	1.017	0.304	0.938

Note: MLE is the mean of the 1000 point estimates via the EM algorithm; width and CP are the average width and coverage proportion of 1000 bootstrap confidence intervals.

**TABLE 2:** MLEs and bootstrap confidence intervals of parameters when m=3

	n=100			n=300			n=500		
True value	MLE	Width	CP	MLE	Width	CP	MLE	Width	CP
$p_{1}$ = 0.1	0.100	0.118	0.936	0.100	0.069	0.940	0.100	0.054	0.953
$p_{2}$= 0.3	0.302	0.180	0.950	0.300	0.104	0.943	0.300	0.081	0.950
$p_{3}$= 0.2	0.198	0.157	0.943	0.199	0.091	0.938	0.199	0.071	0.938
$\alpha_{1}$= 3	3.091	1.528	0.926	3.019	0.840	0.952	3.011	0.646	0.948
$\alpha_{2}$ = 2	2.046	1.033	0.915	2.010	0.572	0.950	2.007	0.440	0.940
$\alpha_{3}$ = 4	4.115	2.069	0.924	4.028	1.143	0.943	4.012	0.879	0.939
$p_{1}$ = 0.2	0.201	0.160	0.953	0.200	0.093	0.948	0.200	0.072	0.955
$p_{2}$= 0.2	0.198	0.158	0.953	0.199	0.092	0.959	0.199	0.072	0.949
$p_{3}$= 0.3	0.299	0.180	0.954	0.300	0.105	0.948	0.300	0.081	0.952
$\alpha_{1}$= 2	2.067	1.088	0.921	2.020	0.595	0.941	2.012	0.456	0.957
$\alpha_{2}$ = 1	1.031	0.514	0.912	1.012	0.284	0.930	1.007	0.218	0.934
$\alpha_{3}$ = 3	3.100	1.696	0.925	3.032	0.930	0.939	3.019	0.710	0.945

Note: MLE is the mean of the 1000 point estimates via the EM algorithm; width and CP are the average width and coverage proportion of 1000 bootstrap confidence intervals.

From Tables 1 and 2, it is clear that the performance of the MLEs is satisfactory in the sense that (i) the bias of the estimate is negligible; (ii) the confidence width is acceptable; and (iii) the coverage probability is from 0.923 to 0.966, which is not far from the prespecified value 1−0.05=0.95. Though 0.923 is a little far from 0.95, the coverage proportion can be improved by increasing the sample size.

### 6.2 Numerical results for the regression model

In this subsection, we conduct simulation to evaluate the performance of the proposed regression model for target parameters. Here, we set m=3 and the regression coefficient vector is $\left(\beta_{1},\beta_{2},\beta_{3},\gamma_{1},\gamma_{2},\gamma_{3}\right)$ with the true values being set and reported in Table 3. We generate ${\left\{x_{i}\right\}}_{i=1}^{500}∼\text{DCD}\left(p,\alpha \right)$, where $p_{ij}=\frac{e^{v_{i}^{⊤}\beta_{j}}}{1+e^{v_{i}^{⊤}\beta_{j}}}$ and $\alpha_{ij}=e^{w_{i}^{⊤}\beta_{j}}$. For each observed data $y_{i}$, we calculate the MLEs $\left({\hat{\beta }}_{1},{\hat{\beta }}_{2},{\hat{\beta }}_{3},{\hat{\gamma }}_{1},{\hat{\gamma }}_{2},{\hat{\gamma }}_{3}\right)$ and this process is repeated 1000 times. The mean value, standard deviation and bootstrap confidence interval are presented in Table 3. According to the results, the MLEs and bootstrap confidence intervals perform well.

**TABLE 3:** MLEs for the regression coefficients in the DCD regression model

Parameter	True	MLE	Width	CP	True	MLE	Width	CP
	0.2	0.211	0.587	0.946	−1	−1.019	0.579	0.915
$\beta_{1}$	−2	−2.022	0.788	0.950	2	2.030	0.709	0.901
	1	1.013	0.594	0.928	−1	−1.012	0.600	0.972
	1	1.019	0.581	0.967	3	3.063	1.244	0.967
$\beta_{2}$	−1	−1.016	0.603	0.939	1	1.029	0.713	0.958
	2	2.031	0.700	0.953	−2	−2.037	0.971	0.944
	−1	−1.019	0.721	0.960	−1	−1.016	0.773	0.923
$\beta_{3}$	−3	−3.059	1.014	0.912	−2	−2.035	1.019	0.918
	3	3.059	1.090	0.936	3	3.047	1.313	0.951
	−1	−0.925	0.357	0.878	−1	−0.986	0.364	0.928
$\gamma_{1}$	−1	−1.012	0.335	0.924	1	0.971	0.338	0.895
	2	1.975	0.291	0.886	−2	−1.963	0.385	0.919
	−2.5	−2.391	0.312	0.862	−2	−1.992	0.345	0.943
$\gamma_{2}$	2	1.894	0.316	0.946	0.5	0.478	0.358	0.922
	−2	−1.888	0.386	0.872	−1.5	−1.472	0.358	0.947
	−1	−0.892	0.377	0.939	−1	−0.993	0.382	0.947
$\gamma_{3}$	1	0.895	0.373	0.959	2	1.974	0.356	0.936
	−2	−1.887	0.341	0.941	−1	−0.973	0.358	0.956

Note: MLE is the mean of the 1000 point estimates via the EM algorithm; width is the mean of the width of the 1000 CIs and CP is the coverage proportion of the confidence intervals.

### 6.3 The sensitivity and robustness of the model

In this subsection, we will investigate the sensitivity and robustness of our model and compare the performance between the DCD and ZIGDM models. Let $u={\left(u_{1},\text{…},u_{m}\right)}^{\;⊤\;}$ be the observation in the ZIGDM model. It is easy to see that the MLEs for the parameters based on u and $u^{\prime }=cu$ with c being any nonzero constant are different under the ZIGDM model. However, the MLE for the parameters under our proposed DCD model will be invariant for constant multiplication. From this perspective, our model is more robust than the ZIGDM model. Next, we will consider the sensitivity of our model. For this purpose, we generate the data from the ZIGDM model and compare the $L_{2}$ distance between the observation and the predictions using the DCD model and ZIGDM model. We set sample size n=100,300,500 and parameters being $\pi_{1}={\left(0,0.1\right)}^{\;⊤\;}$, $\pi_{2}={\left(0.1,0.1\right)}^{\;⊤\;}$, $\pi_{3}={\left(0.2,0\right)}^{\;⊤\;}$, $\pi_{4}={\left(0.2,0.3\right)}^{\;⊤\;}$; $a_{1}={\left(2,3\right)}^{\;⊤\;}$, $b_{1}={\left(5,3\right)}^{\;⊤\;}$, $a_{2}={\left(3,3\right)}^{\;⊤\;}$, $b_{2}={\left(2,4\right)}^{\;⊤\;}$, $a_{3}={\left(4,1\right)}^{\;⊤\;}$, and $b_{3}={\left(2,2\right)}^{\;⊤\;}$. We generate the data ${\left\{u_{i}\right\}}_{i=1}^{n}$ with $u_{i}={\left(u_{i1},\text{…},u_{im}\right)}^{\;⊤\;}$ and each $u_{i}$ following the ZIGDM distribution with $N=N_{1}=\cdots =N_{n}=100$. That is $u_{i}∼\text{ZIGDM}\left(\pi ,a,b\right)$. For ${\left\{u_{i}\right\}}_{i=1}^{n}$, we obtain the MLEs of the parameters $\left(\hat{\pi },\hat{a},\hat{b}\right)$, and then calculate the predictions of the ${\left\{{\hat{u}}_{i}^{\left(1\right)}\right\}}_{i=1}^{n}$ by applying the ZIGDM model. Similarly, the predictions of ${\left\{{\hat{u}}_{i}^{\left(2\right)}\right\}}_{i=1}^{n}$ are obtained based on the DCD model. The $L_{2}$ distance between observations and predictions (denoted as $d_{1}$ and $d_{2}$) are presented in Table 4, where $d_{k}=\sum_{i=1}^{n}{\left(u_{i}-{\hat{u}}_{i}^{\left(k\right)}\right)}^{\;⊤\;}\left(u_{i}-{\hat{u}}_{i}^{\left(k\right)}\right)/N^{2}$ with k = 1 and 2.

**TABLE 4:** The $L_{2}$ distances between observations and predictions: $d_{1}$ and $d_{2}$

		$\pi_{1}$		$\pi_{2}$		$\pi_{3}$		$\pi_{4}$	
Parameters		$d_{1}$	$d_{2}$	$d_{1}$	$d_{2}$	$d_{1}$	$d_{2}$	$d_{1}$	$d_{2}$
$\left(a_{1},b_{1}\right)$	n=100	20.26	20.41	21.50	22.06	20.50	20.40	25.57	27.01
	*n*=300	61.41	61.44	65.31	66.60	62.84	61.61	77.08	81.43
	*n*=500	102.76	102.34	108.60	110.96	104.80	103.00	128.87	135.42
$\left(a_{2},b_{2}\right)$	n=100	16.60	16.11	26.66	26.37	36.53	34.79	31.68	32.87
	n=300	49.94	48.44	81.30	79.34	110.94	105.00	96.05	99.18
	n=500	83.07	80.69	135.23	131.94	184.80	175.47	159.84	165.67
$\left(a_{3},b_{3}\right)$	n=100	14.18	13.79	26.80	26.78	39.48	38.82	32.54	34.27
	n=300	42.66	41.56	81.20	81.19	119.85	117.52	98.16	103.76
	n=500	71.06	69.04	134.98	135.03	200.03	196.08	163.54	172.35

As the data are generated from the DM model, $d_{1}$ is expected to be less than $d_{2}$. From Table 4, $d_{1}$ is generally less than $d_{2}$. It is noticed that the DCD model sometimes fits better than the ZIDGM model. It suggests that our proposed DCD model is robust.

Next, we generate the data from DCD distribution with n=100,300,500 and parameters being $p_{1}={\left(0.4,0.1,0.3\right)}^{\;⊤\;}$, $p_{2}={\left(0,0.1,0.2\right)}^{\;⊤\;}$, $p_{3}={\left(0.2,0,0.3\right)}^{\;⊤\;}$, $p_{4}={\left(0.3,0.2,0.1\right)}^{\;⊤\;}$; $\alpha_{1}={\left(4,5,6\right)}^{\;⊤\;}$, $\alpha_{2}=\left(3,2,1\right){,}^{\;⊤\;}$ and $\alpha_{3}={\left(2,2,5\right)}^{\;⊤\;}$. We obtain the MLEs based on the ZIGDM model and then calculate the $L_{2}$ distance between the predictions and observed data (denoted as $d_{3}$). Since the ZIGDM model does not work for DCD data, we assume $N_{1}=\cdots =N_{m}=100$. Similarly, we can get the $L_{2}$ distance (denoted as $d_{4}$) using the DCD model. We report $d_{3}$ and $d_{4}$ in Table 5. As expected, $d_{3}$ should be generally larger than $d_{4}$. The results in Table 5 support that the performance of the DCD model is generally better than the ZIGDM model.

**TABLE 5:** The $L_{2}$ distances between observations and predictions: $d_{3}$ and $d_{4}$

		$p_{1}$		$p_{2}$		$p_{3}$		$p_{4}$	
Parameters		$d_{3}$	$d_{4}$	$d_{3}$	$d_{4}$	$d_{3}$	$d_{4}$	$d_{3}$	$d_{4}$
$\alpha_{1}$	n=100	40.86	38.72	26.08	21.35	28.90	26.17	30.54	29.37
	*n*=300	122.35	116.52	78.82	64.76	87.35	79.20	92.61	89.08
	*n*=500	204.86	195.15	131.57	107.93	145.43	131.91	154.36	148.83
$\alpha_{2}$	n=100	56.19	52.03	23.50	21.80	36.75	34.89	50.54	50.60
	n=300	169.76	156.88	70.18	65.42	110.42	104.67	152.10	152.17
	n=500	283.58	261.86	117.68	109.50	184.64	174.48	254.46	254.46
$\alpha_{3}$	n=100	52.21	50.98	37.92	31.52	44.45	41.45	34.01	31.12
	n=300	157.61	153.92	113.95	94.95	132.90	124.44	103.05	94.36
	n=500	263.51	257.16	190.19	158.34	223.38	207.44	172.41	157.27

### 6.4 Hypothesis testing

In this subsection, we evaluate the performance of our proposed LRT. We set $\beta_{j}={\left(\beta_{j1},\beta_{j2},\beta_{j3}\right)}^{\;⊤\;}$ and $\gamma_{j}={\left(\gamma_{j1},\gamma_{j2},\gamma_{j3}\right)}^{\;⊤\;}$ for j=1,2,3. According to Equation (8), we first consider Case I:

(32) $$H_{0}:\;\beta_{1}=\beta_{2}=\beta_{3}=0\;\text{against}\;H_{1}:\;H_{0}\;\text{is}\;\text{not}\;\text{true}.$$

We generate the data ${\left\{x_{i}\right\}}_{i=1}^{n}∼\text{DCD}\left(p,\alpha \right)$ for 1000 times, where $\beta_{1}=\beta_{2}=\beta_{3}=0\;\;\;0$ and $\gamma_{1}={\left(-1,-1,2\right)}^{\;⊤\;}$, $\gamma_{2}={\left(-2.5,2,-2\right)}^{\;⊤\;}$, and $\gamma_{3}={\left(-1,1,-2\right)}^{\;⊤\;}$. Second, we consider Case II:

(33) $$H_{0}:\;\gamma_{1}=\gamma_{2}=\gamma_{3}=0\;\;\;0\;\text{against}\;H_{1}:\;H_{0}\;\text{is}\;\text{not}\;\text{true}.$$

We generate the data with $\gamma_{1}^{\prime }=\gamma_{2}^{\prime }=\gamma_{3}^{\prime }=0\;\;\;0$ and $\beta_{1}^{\prime }={\left(0.2,-2,1\right)}^{\;⊤\;}$, $\beta_{2}^{\prime }={\left(0.3,-1,0.5\right)}^{\;⊤\;}$, and $\beta_{3}^{\prime }={\left(-1,-3,3\right)}^{\;⊤\;}$. Third, we consider Case III:

(34) $$H_{0}:\;\beta_{1}=\beta_{2}=\beta_{3}=\gamma_{1}=\gamma_{2}=\gamma_{3}=0\;\text{against}\;H_{1}:\;H_{0}\;\text{is}\;\text{not}\;\text{true}.$$

We generate data with parameters being $\beta_{1}=\beta_{2}=\beta_{3}=\gamma_{1}=\gamma_{2}=\gamma_{3}=0\;\;\;0$. Applying the LRT in Section 5, we record the proportions of rejection of the above three cases with the sample size being n=50, n=100, n=200, n=500, n=1000. The simulated type I error rate is reported in Table 6. It is clearly that the performance of our LRT is fairly good even when the sample size is small. When the sample size is larger than 200, the simulated type I error rate is close to the prespecified significance level (i.e., 0.05).

**TABLE 6:** The type I error rates for the LRT

Type I error	n=50	n=100	n=200	n=500	n=1000
Case I	0.057	0.073	0.057	0.045	0.056
Case II	0.100	0.058	0.040	0.053	0.050
Case III	0.077	0.056	0.047	0.047	0.044

Next, we investigate the power of the LRT. We generate the data from ${\left\{x_{i}\right\}}_{i=1}^{n}∼\text{DCD}\left(p,\alpha \right)$ with parameters being $\left(0\;\;\;0,\beta_{2}^{\prime },0\;\;\;0,0\;\;\;0,\gamma_{2},0\;\;\;0\right)$. The number of rejections according to the above three cases is recorded in Table 7. As expected, the simulated power increases with the sample size.

**TABLE 7:** The simulated powers for the LRT

Type I error	n=50	n=100	n=200	n=500	n=1000
Case I	0.692	0.955	1.000	1.000	1.000
Case II	1.000	1.000	1.000	1.000	1.000
Case III	1.000	1.000	1.000	1.000	1.000

## 7 THE PERCENTAGE OF CHROMOSOME DATA BY THE FISH TEST

In this section, we revisit the FISH test dataset described in Section 1. We here apply the DCD to analyze the FISH data of chromosome 16. First, we analyze the dataset with no covariate, and the results are reported in Table 8.

**TABLE 8:** MLEs and 95% bootstrap CIs of parameters for the parameters of the FISH data

Parameter	MLE	Mean	Median	95% Bootstrap CI
$p_{1}$	0.000	0.000	0.000	[ 0.000, 0.000]
$p_{2}$	0.784	0.774	0.765	[0.667, 0.882]
$p_{3}$	0.980	0.969	0.980	[0.922, 0.980]
$\alpha_{1}$	12.170	10.860	10.435	[6.661, 17.288]
$\alpha_{2}$	38.516	33.009	32.074	[17.123, 54.360]
$\alpha_{3}$	6.117	5.359	5.202	[3.146, 8.399]

As we can see from Table 8, the first component in the composition dataset (i.e., the normal cell) is all nonzeros, ${\hat{p}}_{1}=0$ means that the probability of zero observation for the normal cell is 0. The probability of zero observations of the triple and tetraploid cell is estimated to be 0.784 and 0.980, respectively. That is, for the chromosome 16 the triple cell can be found with 20% percentage while tetraploid can be found with only 2% percentage. For the base part of the compositional data, the estimate corresponding to the triple cell is larger than those of the normal and tetraploid cells; that is, ${\hat{\alpha }}_{1}$ = 12.17, ${\hat{\alpha }}_{1}$ = 38.52, and ${\hat{\alpha }}_{3}$ = 6.12. In other words, once there is abnormal chromosome in the cell the triple is more frequent than the other two.

Due to the assumption of the normal distribution for covariates in the regression model, we make the standardization for covariate age in the FISH dataset. Furthermore, we apply the regression model to investigate the relationship between parameters (p,α) and the age of gravida. The MLEs of the coefficients are reported in Table 9. We apply the LRT to the hypotheses listed in Section 5, and the null hypothesis is rejected at α = 0.05. Therefore, we have reason to believe that age is a significant variable.

**TABLE 9:** MLEs and 95% bootstrap CIs of parameters for the coefficients in the regression model of the FISH data

Parameter	Coefficients	|cMLE	Standard deviation	95% Bootstrap CI
$\beta_{1}$	Intercept	−12.985	2.912	[−19.302, −8.034]
	Age	1.019	1.840	[−2.419, 4.221]
$\beta_{2}$	Intercept	1.293	0.371	[0.663, 2.076]
	Age	0.080	0.388	[−0.680, 0.942]
$\beta_{3}$	Intercept	4.192	0.459	[2.779, 4.448]
	Age	0.796	0.316	[0.458, 1.753]
$\gamma_{1}$	Intercept	3.116	0.559	[1.238, 3.798]
	Age	1.003	0.992	[−2.461, 2.082]
$\gamma_{2}$	Intercept	4.270	0.652	[1.876, 4.904]
	Age	1.178	1.036	[−2.534, 2.234]
$\gamma_{3}$	Intercept	1.234	0.329	[ 0.729, 1.803]
	Age	− 0.615	0.150	[−0.925, −0.366]

## 8 DISCUSSION

In this article, we consider a new framework for analyzing compositional data with zero entries based on SR. In particular, a new distribution, namely the DCD, is developed to accommodate the possible essential‐zero feature in compositional data (i.e., some components are completely absent). In our proposed distribution, the elements in the base vector are assumed to follow independent gamma distributions. Therefore, any positive random variable can be adopted as an element of the base vector (e.g., the inverse Gaussian and chi‐square random variables), and different base vectors will correspond to a different relationship among elements. It is of research and practical interests to relax the assumption of independence among the components in the base vector. Besides, regression modeling for high‐dimensional covariates is also an interesting and necessary topic as the Jacobi tends to be zero when the dimension of covariates is high.

## CONFLICT OF INTEREST

The authors have declared no conflict of interest.

### OPEN RESEARCH BADGES

This article has earned an Open Data badge for making publicly available the digitally‐shareable data necessary to reproduce the reported results. The data is available in the Supporting Information section.

This article has earned an open data badge “**Reproducible Research**” for making publicly available the code necessary to reproduce the reported results. The results reported in this article were reproduced partially due to their computational complexity.

## Supporting information

Supporting Information.

## Data Availability

Data are available on request from the authors.

## Appendix

Before we present the proofs, we first introduce the following two lemmas. Lemma 1 Let $X={\left(X_{1},\text{…},X_{m}\right)}^{⊤}$ follow the composition Dirichlet distribution DCD(p,α), Y be the base vector with $Y_{i}\overset{\mathrm{ind}}{∼}\Gamma \left(\alpha_{i},1\right)$ and Z be an indicator vector with Y⊥⊥Z. If $s=\sum_{i=1}^{m}Z_{i}Y_{i}$, then the joint probability density function of (X,s) conditioned on Z is given by

(A.1)
$$f\left(x,s|z\right)=f\left(x_{J},s\right)=s^{\alpha -1}e^{-s}\times \left(\prod_{j\in J}\frac{x_{j}^{\alpha_{j}-1}}{\Gamma (\alpha_{j})}\right),$$

where J is the subset of the index with X being positive; that is, we have $x_{j}>0$ for j∈J, $x_{j}=0$ for j∉J and $\sum_{j=1}^{m}X_{j}=1$.

Without loss of generality, we assume that the first r elements of X is positive; that is, J={1,2,…,r}. The joint probability density function of $\left(Y_{1},\text{…},Y_{r}\right)$ is then given by

(A.2)
$$f\left(y_{1},\text{…},y_{r}\right)=\prod_{j=1}^{r}\frac{y_{j}^{\alpha_{j}-1}e^{-y_{j}}}{\Gamma (\alpha_{j})}.$$

Next, we consider the following transformation:

(A.3)
$$\begin{array}{c} \left\{\begin{array}{ccc} s & = & \sum_{i=1}^{r}Y_{i}.\\ X_{1} & = & Y_{1}/s,\\ & \vdots & \\ X_{r-1} & = & Y_{r-1}/s. \end{array}\right.⟺\left\{\begin{array}{ccc} Y_{1} & = & sX_{1},\\ & \vdots & \\ Y_{r-1} & = & sX_{r-1},\\ Y_{r} & = & s(1-\sum_{i=1}^{r-1}X_{i}). \end{array}\right. \end{array}$$

Obviously, $Y_{r}={\left(Y_{1},\text{…},Y_{r}\right)}^{⊤}$ and ${\left(s,X_{r-1}\right)}^{⊤}={\left(s,X_{1},\text{…},X_{r-1}\right)}^{⊤}$ are one‐to‐one transformations. The Jacobian determinant from $Y_{r}$ to $\left(s,X_{r-1}\right)$ is given by

(A.4)
$$\begin{array}{ccc} J(y_{r}|s,X_{r-1}) & = & \left|\begin{array}{ccccc} X_{1} & s & 0 & \cdots & 0\\ X_{2} & 0 & s & \cdots & 0\\ \vdots & 0 & 0 & \ddots & 0\\ X_{r-1} & 0 & 0 & \cdots & s\\ 1-\sum_{i=1}^{r-1}X_{i} & -s & -s & \cdots & -s \end{array}\right|\\ & = & \left|\begin{array}{ccccc} X_{1} & s & 0 & \cdots & 0\\ X_{2} & 0 & s & \cdots & 0\\ \vdots & 0 & 0 & \ddots & 0\\ X_{r-1} & 0 & 0 & \cdots & s\\ 1 & 0 & 0 & \cdots & 0 \end{array}\right|=s^{r-1}. \end{array}$$

The probability density function of $\left(s,X_{r-1}\right)$ given Z is then given by

(A.5)
$$f\left(s,x_{r-1}|z\right)=f\left(y_{r}\right)J\left(y_{r}|s,x_{r-1}\right)=s^{r-1}\prod_{j=1}^{r}\frac{{\left(sx_{j}\right)}^{\alpha_{j}-1}e^{-sx_{j}}}{\Gamma (\alpha_{j})}=s^{\alpha_{J}^{*}-1}e^{-s}\prod_{j=1}^{r}\frac{x_{j}^{\alpha_{j}-1}}{\Gamma (\alpha_{j})},$$

where $\alpha_{J}^{*}=\sum_{j=1}^{r}\alpha_{j}$, $x_{r}=1-\sum_{i=1}^{r-1}x_{i}$.□

Lemma 2 Let $X={\left(X_{1},\text{…},X_{m}\right)}^{⊤}$ follow the composition Dirichlet distribution DCD(p,α), Y be the base vector with $Y_{i}\overset{\mathrm{ind}}{∼}\Gamma \left(\alpha_{i},1\right)$ and Z be an indicator vector with Y⊥⊥Z. If $s=\sum_{i=1}^{m}Z_{i}Y_{i}$, then the probability density function of X conditioned on Z is given by

(A.6)
$$f\left(x|z\right)=f\left(x_{J}\right)=\Gamma \left(\alpha_{J}^{*}\right)\prod_{j\in J}\frac{x_{j}^{\alpha_{j}-1}}{\Gamma (\alpha_{j})}=\Gamma \left(\alpha_{J}^{*}\right)\prod_{j\in J}\frac{x_{j}^{\alpha_{j}-1}}{\Gamma (\alpha_{j})},$$

where $\alpha_{J}^{*}=\sum_{j=1}^{r}\alpha_{j}$.

(A.7)
$$f\left(x_{J}\right)=\int_{0}^{+\infty }f\left(s,x_{J}\right)ds=\int_{0}^{+\infty }s^{\alpha_{J}^{*}-1}e^{-s}\prod_{j\in J}\frac{x_{j}^{\alpha_{j}-1}}{\Gamma (\alpha_{j})}ds=\Gamma \left(\alpha_{J}^{*}\right)\prod_{j\in J}\frac{x_{j}^{\alpha_{j}-1}}{\Gamma (\alpha_{j})}.$$

□

### A.1 Proof of the pdf of DCD

The probability density function of the DCD is

(A.8)
$$\begin{array}{c} f\left(x\right)=\frac{\prod_{j=1}^{m}p_{j}^{1-z_{j}}{\left(1-p_{j}\right)}^{z_{j}}}{1-p_{1}\cdots p_{m}}\times \left[\frac{\Gamma (\alpha_{J_{i}}^{*})}{\prod_{{}_{j\in J}}\Gamma \left(\alpha_{j}\right)}\prod_{{}_{j\in J}}x_{j}^{\alpha_{j}-1}\right]. \end{array}$$

The pdf of the DCD can be derived via its SR; that is, $X\overset{d}{=}\frac{Z\circ Y}{\left(Z,Y\right)}$. Actually, the indicator vector z is derived from x by $z_{j}=I\left(x_{j}>0\right)$, j=1,…,m. It is clear that the indicator vector Z is determined by the compositional data X, the conditional mass function of Z is $\mathrm{Pr}\left(Z_{1}=z_{1},\text{…},Z_{m}=z_{m}|x\right)=I\left(z_{1}=I\left(x_{1}>0\right),\text{…},z_{m}=I\left(z_{m}>0\right)\right)$. Thus we have f(x,z)=f(z|x)f(x)=f(x)
Therefore, the probability density function (pdf) of DCD is

(A.9)
$$\begin{array}{c} f\left(x\right)=f\left(x,z\right)=f\left(x|z\right)f\left(z\right)=\frac{\Gamma (\alpha_{J_{i}}^{*})}{\prod_{{}_{j\in J}}\Gamma \left(\alpha_{j}\right)}\prod_{{}_{j\in J}}x_{j}^{\alpha_{j}-1}\times \frac{\prod_{j=1}^{m}{\left(1-p_{j}\right)}^{z_{j}}p_{j}^{1-z_{j}}}{1-p_{1}\cdots p_{m}}. \end{array}$$

□

### A.2 Proof of Theorem 1

Lemma 3 Let $X={\left(X_{1},\text{…},X_{m}\right)}^{⊤}$ follow the composition Dirichlet distribution DCD(p,α), Y be the base vector with $Y_{i}\overset{\mathrm{ind}}{∼}\Gamma \left(\alpha_{i},1\right)$ and Z be an indicator vector with Y⊥⊥Z. If $s=\sum_{i=1}^{m}Z_{i}Y_{i}$, then the joint probability density function of $\left(Y_{j},X_{J}\right)$ is given as follows:

(A.10)
$$f\left(y_{j},x_{J}\right)=f\left(s,x_{J}\right)\frac{1}{x_{j}}=\frac{1}{x_{j}}s^{\alpha_{J}^{*}-1}e^{-s}\prod_{j\in J}\frac{x_{j}^{\alpha_{j}-1}}{\Gamma (\alpha_{j})},$$

where j∈J, $\alpha_{J}^{*}=\sum_{j=1}^{r}\alpha_{j}$, $s=y_{j}/x_{j}$.

The proof follows immediately by using the transformation $s=y_{j}/x_{j}$.□

Lemma 4 Let $X={\left(X_{1},\text{…},X_{m}\right)}^{⊤}$ follow the composition Dirichlet distribution DCD(p,α), Y be the base vector with $Y_{i}\overset{\mathrm{ind}}{∼}\Gamma \left(\alpha_{i},1\right)$ and Z be an indicator vector with Y⊥⊥Z. If $s=\sum_{i=1}^{m}Z_{i}Y_{i}$, then we have

(A.11)
$$\begin{array}{c} E\left(\log y_{j}|x_{j}>0\right)=\psi \left(\alpha_{J_{i}}^{*}\right)+\log x_{j}, \end{array}$$

where J is the subset of the index with x being positive; that is, for any j∈J, we have $x_{j}>0$; otherwise $x_{j}=0$.

(A.12)
$$\begin{array}{ccc} E(\log y_{j}|x) & = & \int_{0}^{+\infty }\log y_{j}f\left(y_{j}|x\right)dy_{j}=\int_{0}^{+\infty }\log y_{j}\times \frac{\frac{1}{x_{j}}s^{\alpha_{J}^{*}-1}e^{-s}\prod_{j\in J}\frac{x_{j}^{\alpha_{j}-1}}{\Gamma (\alpha_{j})}}{\Gamma \left(\alpha_{J}^{*}\right)\prod_{j\in J}\frac{x_{j}^{\alpha_{j}-1}}{\Gamma (\alpha_{j})}}dy_{j}\\ & \overset{\left(2\right)}{=} & \int_{0}^{+\infty }\frac{\left(\log s+\log x_{j}\right)e^{-s}s^{\alpha_{J}^{*}-1}}{\Gamma (\alpha_{J}^{*})}ds\\ & = & \int_{0}^{+\infty }\frac{\left(\log s\right)\times e^{-s}s^{\alpha_{J}^{*}-1}}{\Gamma (\alpha_{J}^{*})}ds+\int_{0}^{+\infty }\log x_{j}\times \frac{e^{-s}s^{\alpha_{J}^{*}-1}}{\Gamma (\alpha_{J}^{*})}ds\\ & = & \psi \left(\alpha_{J}^{*}\right)+\log x_{j}. \end{array}$$

Note: We make the transformation $y_{j}=sx_{j}$ above.□

Lemma 5 Let $X={\left(X_{1},\text{…},X_{m}\right)}^{⊤}$ follow the composition Dirichlet distribution DCD(p,α), Y be the base vector with $Y_{i}\overset{\mathrm{ind}}{∼}\Gamma \left(\alpha_{i},1\right)$ and Z be an indicator vector with $Z_{i}∼\text{Bernoulli}\left(1-p_{i}\right)$ and Y⊥⊥Z. If $s=\sum_{i=1}^{m}Z_{i}Y_{i}$, then we have

(A.13)
$$E\left(\log y_{j}|x_{j}=0\right)=\psi \left(\alpha_{j}\right).$$

(A.14)
$$\begin{array}{ccc} E(\log y_{j}|x_{j}=0) & = & E\left(\log y_{j}|z_{j}=0\right)=E\left(\log y_{j}\right)\\ & = & \int_{0}^{+\infty }\log y_{j}f\left(y_{j}\right)dy_{j}=\int_{0}^{+\infty }\log y_{j}\times \frac{y_{j}^{\alpha_{j}-1}e^{-y_{j}}}{\Gamma (\alpha_{j})}dy_{j}\\ & = & \psi (\alpha_{j}). \end{array}$$

Based on the results in Lemmas 4 and 5, Theorem 1 follows immediately with

(A.15)
$$E\left(\log y_{j}|x\right)=\left\{\begin{array}{ccc} \psi (\alpha_{j}), & & \text{if}\;x_{j}=0,\\ \psi \left(\alpha_{J_{i}}^{*}\right)+\log x_{j} & & \text{if}\;x_{ij}>0. \end{array}\right.$$

□
